# New Methods of Treatment in Phthisis

**Published:** 1891-10-24

**Authors:** 


### The Practitioner's Mirror and Retrospect.

[*The Editor will be glad to receive offers of co-operation and contributions from members of the profession. All letters should be addressed to* The Editor, The Lodge, Porchester Square, London, W.]

### MEDICINE.

### NEW METHODS OF TREATMENT IN PHTHISIS.

IV.—Transfusion of the Blood of Refractory Animals.

As yet we believe that only two animals have been put forward as capable of being utilised for the salvation of tuberculous human beings by the transfusion of their blood serum, viz., the goat and the dog, the assumption being that their insusceptibility to phthisis and tuberculosis generally is due to their blood containing some constituent which is fatal to the growth of the bacillus.

As the goat serum method was, we believe, first in the field, we will allude to this first. It was introduced by Dr. Lépine, of Lyons. He found that the subcutaneous injections of large quantities of the serum rendered the site of injection very painful, he, therefore, adopted intravenous injection as the most suitable method of procedure. He found eight to a hundred cubic centimeters might be injected at a time, and repeated every two or three days. There was practically no danger if proper precautions were observed, and no pain. Dr. Lepine uses the blood of the goat caught in a basin, defibrinated, and the corpuscles removed by rapid rotation of a disc on which are placed tubes. The purified serum is then transferred to a sterilised vessel provided with an india-rubber tube issuing from the bottom and closed above with cotton wool. The patient's arm having been disinfected and tied as for phlebotomy, a prominent vein is selected and a very fine silver trochar inserted without dissection, which is by no means a difficult operation. This is cautiously connected with the india-rubber tube, and the serum allowed to flow into the vein very slowly. If the patient should feel malaise, the flow must be stopped by lowering the reservoir. While 100 c.cm. of the serum could be injected at once, not more than 40 grammes of the blood itself could be borne, the patients complaining for some inexplicable reason of a pain in the loins. At a meeting of the Paris Société de Therapeutique, Dr. Bernheim brought forward his results in a large number of experiments on animals, and from some observations made on patients suffering from phthisis and chlorosis, he said he felt justified in drawing the following conclusions: The transfusion of goats' blood is in no way dangerous if properly performed; several tuberculosis subjects were so far improved that they might be looked upon as cured. M. Bertin and Picq have obtained some good results in fifty cases in which they had employed this method. These included seventeen cases of internal tuberculosis, and in eleven of these, great improvement followed the treatment. Temperature fell, night sweats and expectoration ceased or diminished to a marked degree, physical signs cleared up, appetite was recovered, and weight was gained. In two cases the injections caused abscesses; in one which was complicated with lupus, the latter condition was much improved, but nothing is said as to the pulmonary affection; two patients had died, but in each of these the disease was far advanced before the treatment was begun. Of five cases of external tuberculosis (sacrum, bones of the hand and leg, hip joint, with resection of the upper ends of the femur, Potts' disease, bones of the foot with affection of the lung), the improvement was not so marked, but yet the general condition was improved, while locally there was diminution of suppuration, and, in some cases, healing. Now as regards the injection of dog's serum, we must remember that the insusceptibility of the dog to tuberculosis is only a question of degree. It is ar less susceptible than human beings, and very much more insusceptible than the rabbit. Yet we must remember that tuberculosis in the dog is not unknown. At a meeting of the Société de Biologie, in the month of January, Roget brought forward some specimens from a dog that had become affected by tuberculous meat, and had died of pulmonary tuberculosis. The pleura of the animal was very much thickened, and in the lung was a nodule containing bacilli resembling the Koch bacillus in reaction to staining reagents, though longer and more attenuated than those found in human phthisical subjects. M. Feulard has recorded his results in ten cases in which this method was adopted. The majority were suffering from lupus, but three or four were cases of syphilitic disease. The injections of at first two c.cm., and afterwards of one c.cm., were given every two days, under the strictest antiseptic precautions. Of a total of two hundred and forty injections, only one was followed by abscess. The treatment was continued in some cases for four months, and in others for a few weeks only. In all of them weight was increased to a considerable amount. In three of the lupus cases, the local condition showed material improvement, although no local treatment had been applied. In one case of syphilitic lesions of the most obstinate nature, the ulcers healed in a short time under the injections, without any other medication, but during the treatment there was a fresh outbreak of syphilides, when iodide of potassium was administered, together with the serum treatment, and a cure was effected. They referred to the experiments of Richet and Héricourt, which have shown that serum has no direct curative action in tuberculosis when fairly established, but that it acts as a powerful general tonic, giving the organism increased strength to resist the progress of the disease, and their clinical experience, they claim, has borne out these laboratory results. Professor Semmola has also testified to his beneficial experience of these injections in phthisis. His observations were based on its employment in ten cases. Semmola assumed that the dog, unlike the goat, was absolutely insusceptible to tuberculosis. This, however, is disproved by the case we have referred to above. In four of his cases the disease was advanced. The dose injected was about one to two c.c.m. daily. In two cases in which there was extensive excavation no benefit followed, nor was there any improvement in a third, in which, although the lesions were not very marked, the temperature was high. In the seven other cases the results were very encouraging. The course of the disease was absolutely modified, the fever subsiding in most cases, and weight being gained in all of three. In three the physical signs disappeared almost entirely, expectoration diminished, and the bacilli decreased in number, or else disappeared altogether. Professor Semmola urged on the Academy the desirability of testing the method by clinical experiments on a vast scale.

We recently referred to the new cure for snake bite advocated by the Municipal Commissioner of Baroda, which was based on the well-known fact that the common weazel is insusceptible to snake bite. It was assumed that this insusceptibility arose from the blood of the weasel containing some substance which acted on the snake poison and rendered it harmless, and which substance is innocuous to human beings inasmuch as the weasel is commonly eaten by the natives. It was suggested that the injection of weasels' blood into human beings who have been bitten would likewise render them insusceptible. But, in reference to this weasel blood cure for snake bite, and the goat blood cure for tuberculosis, we ventured to say that granted that the weasel and the goat are insusceptible to the virus of snake venom and the tubercle bacillus respectively, there is every reason to believe that such insusceptibility is due to the tissues not being acted on by the virus, rather than due to the presence of any antidote in the blood, and that this insusceptibility was due to the modifications in chemical constitution of the tissues, and that the immunity of these animals might be compared to the immunity of certain higher vertebrata to poisonous herbs.

References.—*The Gazette*, March 16th, 1891. *British Medical Journal (Supplement)*, August 22nd, 1891; June 20th, 1891. *Gaz. Méd. de Nantes*, April 12th. *Semaine Med.*, July 15th, 1891. *Gazz. Med. Lomb.*, July 4th, 1891.

